# Editorial: Radiomics and AI-driven deep learning for cancer diagnosis and treatment

**DOI:** 10.3389/fonc.2026.1979773

**Published:** 2026-09-21

**Authors:** Arka Bhowmik, Sunitha B. Thakur, Almir G. V. Bitencourt, Katja Pinker

**Affiliations:** 1 Department of Radiology, Memorial Sloan Kettering Cancer Center, New York, NY, United States; 2 Department of Medical Physics, Memorial Sloan Kettering Cancer Center, New York, NY, United States; 3 Imaging Department, A.C. Camargo Cancer Center, São Paulo, Brazil; 4 Department of Radiology, Columbia University, New York, NY, United States

**Keywords:** auto-segmentation, CT, diagnosis, habitat analysis, histology, MRI, PET, prognosis

Radiomics, habitat clustering, and hybrid approaches are increasingly being investigated as methods for extracting quantitative features from medical images. These features are often integrated with machine learning (ML) and deep learning (DL) models to develop predictive tools that may outperform conventional visual interpretation of medical images. In oncology, their potential applications include tumor detection and characterization, pathological and risk assessment, treatment planning, and longitudinal evaluation of therapeutic outcomes. However, the translation of these methods into routine clinical practice remains constrained by labor-intensive segmentation and preprocessing, variability across imaging platforms and institutions, limited generalizability and external validation, and challenges associated with integration into clinical workflows.

This research topic brings together 19 research articles spanning multiple cancer types (*see* **Figure 1**) and imaging modalities, with particular emphasis on the use of quantitative imaging for multimodal integration, tumor heterogeneity analysis, automated image analysis, independent validation, and clinically relevant prediction. It also includes one opinion article demonstrating the use of a commercial DL-based tool for lung cancer detection from chest radiograph (Zavaleta-Monestel et al.) and one systematic review (Lu et al.) focused on the preoperative differentiation of pancreatic cancer from pancreatitis. Major themes across these studies include the use of intratumoral and/or peritumoral features, often in combination with multimodal imaging, to improve lesion characterization and to predict adverse pathological findings, recurrence, treatment response, and treatment-related risks. Several studies also illustrate a broader shift toward automation, multimodal data fusion, and clinically scalable artificial intelligence (AI) pipelines, including applications to histological images and the integration of histopathology with RNA sequencing (RNA-seq) data.

**Figure 1:**
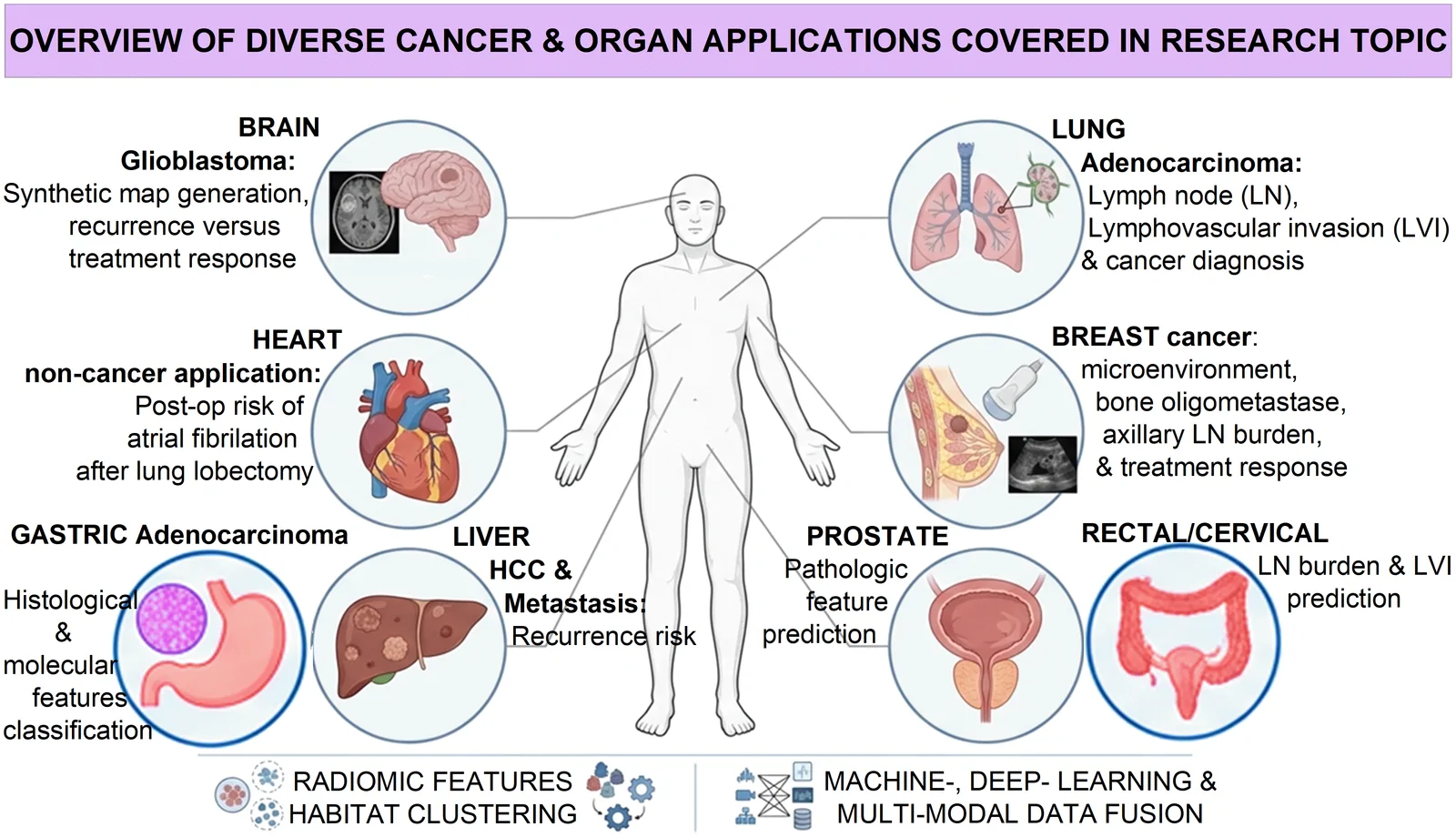
Schematic diagram of diverse cancer and organ applications covered in this research topic. Quantitative feature extraction methodologies (habitat clustering and radiomics) from MRI, CT, PET, Ultrasound, or histopathology images are integrated with artificial intelligence models (machine learning, deep learning, or multimodal data fusion) across multi-organ sites (brain, heart, lung, breast, stomach, liver, prostate, and cervix/rectum) to evaluate tumor heterogeneity, pathological risk features, treatment response, and patient outcomes.

## Pretreatment and presurgical risk stratification

In this research topic, one prominent theme is the use of radiomics and/or habitat-clustering-based ML models to obtain clinically important risk information prior to treatment or surgery. In rectal cancer, Qubie et al. developed T_2_-weighted (T_2_w) MRI-based radiomic models for distinguishing lower (N0/N1) from higher (N2) lymph node (LN) burden in stage T3 rectal cancer, with logistic regression outperforming conventional image assessment. A related clinical objective was addressed in breast cancer by Xu et al., whose study combined B-mode ultrasound and shear-wave elastography (SWE) with habitat-cluster radiomic features to characterize axillary LN burden. A strong performance was achieved using the SWE habitat model, indicating that spatially resolved heterogeneous habitat clustering may contain information not captured by whole-tumor radiomics. In lung cancer, Wumener et al. integrated features extracted from CT, static and dynamic ^18^F-FDG PET images with ML models to improve discrimination between metastatic and benign FDG-avid LN. Collectively, these studies illustrate how quantitative imaging may refine LN assessment in settings where conventional morphology or metabolic activity alone is insufficiently specific.

The same principle extends to prognostic features that may otherwise require invasive assessment or postoperative determination. Zhou et al. combined multiphasic CT radiomics with routine preoperative clinical variables (e.g., age, proteinuria, hematuria, and tumor diameter) to preoperatively distinguish high- from low-grade bladder urothelial carcinoma. Jiang et al. demonstrated that a hybrid model integrating biparametric MRI-based radiomic features derived from apparent diffusion coefficient (ADC) and T_2_w images with preoperative clinical variables, including biopsy-derived Gleason grade, serum prostate-specific antigen (PSA), and prostate volume, improved the prediction of adverse pathological features in prostate cancer compared with either component alone. In cervical cancer, Li et al. performed habitat clustering to identify functional intratumoral subregions followed by radiomic feature extraction, all from amide proton transfer-weighted MRI. These features were subsequently used to train an ML model for predicting lymphovascular invasion (LVI). Notably, incorporating non-invasive clinical and radiological variables, specifically serum CA125 and tumor size, into the habitat-based model further improved predictive accuracy (Li et al.). Similarly, Peng et al. integrated intratumoral radiomic and deep-learning features extracted from CT images into a multilayer perceptron (MLP), a type of neural network that combines multiple interconnected layers where each layer consist of perception elements known as neurons to learn non-linear patterns in data, for LVI prediction in T1-stage invasive lung adenocarcinoma, achieving robust performance across multicenter retrospective datasets. Zhao et al. further demonstrated the potential of DL models based on cropped contrast-enhanced CT images for risk classification of thymic epithelial tumors, with one DL model outperforming an intratumoral radiomics-based ML model during external testing. Importantly, DL assistance also improved the performance of junior radiologists, highlighting a potential role for these models as clinical decision-support tools rather than as fully autonomous diagnostic systems (Zhao et al.).

## Spatial heterogeneity and peritumoral features

Several contributions emphasize that the biological information captured by medical images is not confined to intratumoral features. Kim et al. prospectively evaluated ML models based on intratumoral and peritumoral MRI radiomic features for characterizing extracellular matrix composition and immune-cell abundance within the tumor microenvironment of invasive breast cancer. Models combining intratumoral and peritumoral radiomic features generally improved characterization, particularly when features were derived from postcontrast T1-weighted (T_1_w) images (Kim et al.). This finding complements the habitat-clustering approaches of Xu et al. and Li et al., in which tumors were partitioned into spatially distinct subregions rather than treated as homogeneous volumes. Collectively, these studies reinforce the value of explicitly modeling spatial heterogeneity. However, differences in habitat construction, definitions of peritumoral regions, imaging sequences, and preprocessing methods will need to be standardized or systematically addressed before such imaging signatures can be reliably compared across institutions.

## Multimodal data integration

Multimodal integration represents another approach adopted by researchers to achieve richer representations of tumor biology. Lu et al. combined intratumoral radiomic features, cropped region-of-interest (ROI)-based deep-learning features, model-derived metabolic parameters from PET/CT images, and clinical parameters for the early prediction of bone oligometastases in patients with breast cancer. The integrated multimodal models outperformed visual PET/CT assessment within the study cohort. Extending beyond radiological imaging alone, Wang et al. fused histopathological image features with differentially expressed genes derived from RNA-seq data to classify microsatellite instability versus microsatellite stability in patients with gastric adenocarcinoma. The performance differences between pathology image-only and molecular-based models, together with the improved performance of the multimodal deep-learning fusion model, illustrate the added value of integrating complementary molecular information. Additionally, Zhou et al. developed DL models that integrated pancreatic CT images with encoded clinical text, alongside a semi-supervised framework for pancreas and pancreatic tumor segmentation. Collectively, these studies extend multimodal modeling beyond the simple concatenation of imaging sequences toward the integrated fusion of imaging, molecular, pathological, and semantic information.

## Post-treatment monitoring and recurrence prediction

Several contributions also demonstrate how ML- and DL-based methods can address clinically relevant events following treatment. In hepatocellular carcinoma (HCC), Wang et al. used handcrafted intratumoral radiomic features and cropped region-based DL features extracted from preoperative MRI to predict early peritumoral recurrence after drug-eluting bead transarterial chemoembolization. The combined ML model integrating radiomic and DL features outperformed models based on handcrafted radiomic features alone and DL models using combined features. Wu et al. addressed a different post-treatment challenge by synthesizing cerebral blood volume (CBV) maps from arterial spin labeling (ASL) MRI and standard MRI sequences, including T_1_w, T_2_w, ADC, and contrast-enhanced T_1_w images, using a generative DL algorithm. When combined with standard MRI, the synthetic CBV maps improved differentiation between glioblastoma recurrence and treatment response compared with ASL alone, suggesting that generative DL models may provide clinically useful surrogate images when conventional imaging is technically constrained. In patients with colorectal cancer liver metastases, Lin et al. found that combining CT-derived radiomic features with clinical variables in an ML model yielded a modest improvement in predicting early recurrence within one year after surgery. The study also highlighted limitations associated with a small cohort and the absence of external validation. In breast cancer treatment monitoring, Malherbe et al. prospectively evaluated a DL system designed for real-time risk stratification and treatment-response monitoring in HER2-positive breast cancer using B-mode ultrasound. The investigators observed trends toward decreasing DL-derived malignancy-risk scores during treatment response across two systemic therapy regimens, trastuzumab (Herceptin) and doxorubicin (Stradexa), supporting the exploratory feasibility of DL-assisted ultrasound as a low-cost adjunct for treatment monitoring.

## Patient-level phenotyping beyond the tumor

Clinically relevant radiomic features and imaging biomarkers need not be tumor-focused. They can also provide valuable information for patient-level phenotyping. In lung cancer surgery, Liu et al. integrated clinical factors with radiomic and DL features derived from preoperative CT images of epicardial adipose tissue into an ML model to identify patients at high risk of postoperative atrial fibrillation following lung lobectomy. External validation supported the added value of this imaging-based representation, although the low event rate remained an important limitation (Liu et al.). Similarly, in a retrospective study, Delaye et al. used DL-based software to extract anthropometric parameters, including skeletal muscle mass, total adipose tissue, subcutaneous adipose tissue, visceral adipose tissue, and lean body mass, from three-dimensional CT images to evaluate the association between patient body composition and survival and treatment-related toxicity in patients receiving antibody–drug conjugates in early-phase clinical trials.

## Automation, reproducibility, and clinical integration

Automation and reproducibility are central to translating ML- and DL-based methods into practical clinical applications. Han et al. developed an automated DL pipeline integrating tumor localization, segmentation, cell detection, and calculation of the programmed cell death ligand-1 (PD-L1) combined positive score (CPS) in whole-slide immunohistochemistry images of gastric cancer. The system demonstrated strong agreement with pathologists in both internal and external cohorts. However, residual discrepancies related to staining variability and imaging artifacts suggest that, at its current stage, the tool is better positioned as an assistive system rather than a replacement for standard manual PD-L1 CPS assessment. This work, together with the automated segmentation strategy of Zhou et al, the AI-assisted interpretation evaluated by Zhao et al., and the flexible image-synthesis framework developed by Wu et al., illustrates a broader transition from isolated predictive algorithms toward integrated systems designed to reduce manual workload and facilitate implementation within clinical workflows.

## Conclusions and future directions

Taken together, the studies assembled in this research topic demonstrate that habitat clustering, radiomics, ML-, and DL-based models can interrogate multiple dimensions of cancer care, ranging from tumor and LN characterization to biological phenotyping, treatment response assessment, recurrence risk prediction, and treatment-related complications. Their collective contribution lies not in identifying a single optimal algorithm, but rather in demonstrating complementary strategies for extracting clinically relevant information from diverse imaging and non-imaging data.

However, important methodological limitations persist across the field. Many studies remain retrospective and single-center, and several rely on manual segmentation and ROI selection, institution-specific preprocessing, modest sample sizes, or lack external validation. Even among multicenter studies, model performance sometimes declined when evaluated on external datasets. Therefore, the next phase of progress will depend on translating promising predictive performance into systems that are reproducible, generalizable, interpretable, and efficient to deploy in clinical practice. Advances in automated segmentation, multimodal modeling, external and prospective validation, and the use of AI as an adjunct to human decision-making represent particularly important directions for narrowing the remaining gap between methodological innovation and routine clinical implementation.

